# Supplementary material for: The interactome of intact mitochondria by cross-linking mass spectrometry provides evidence for coexisting respiratory supercomplexes
Source: Mol Cell Proteomics. 2017 Dec 8;17(2):216–32. doi: 10.1074/mcp.RA117.000470 (PMC5795388; doi:10.1074/mcp.RA117.000470)
Supplement: Supplemental Data [file supp_RA117.000470_133922_0_supp_23978_fzffwf.zip › spectra_annotation/mito_DR_spectra_annotation/270-1-1-1-5-1.pdf]

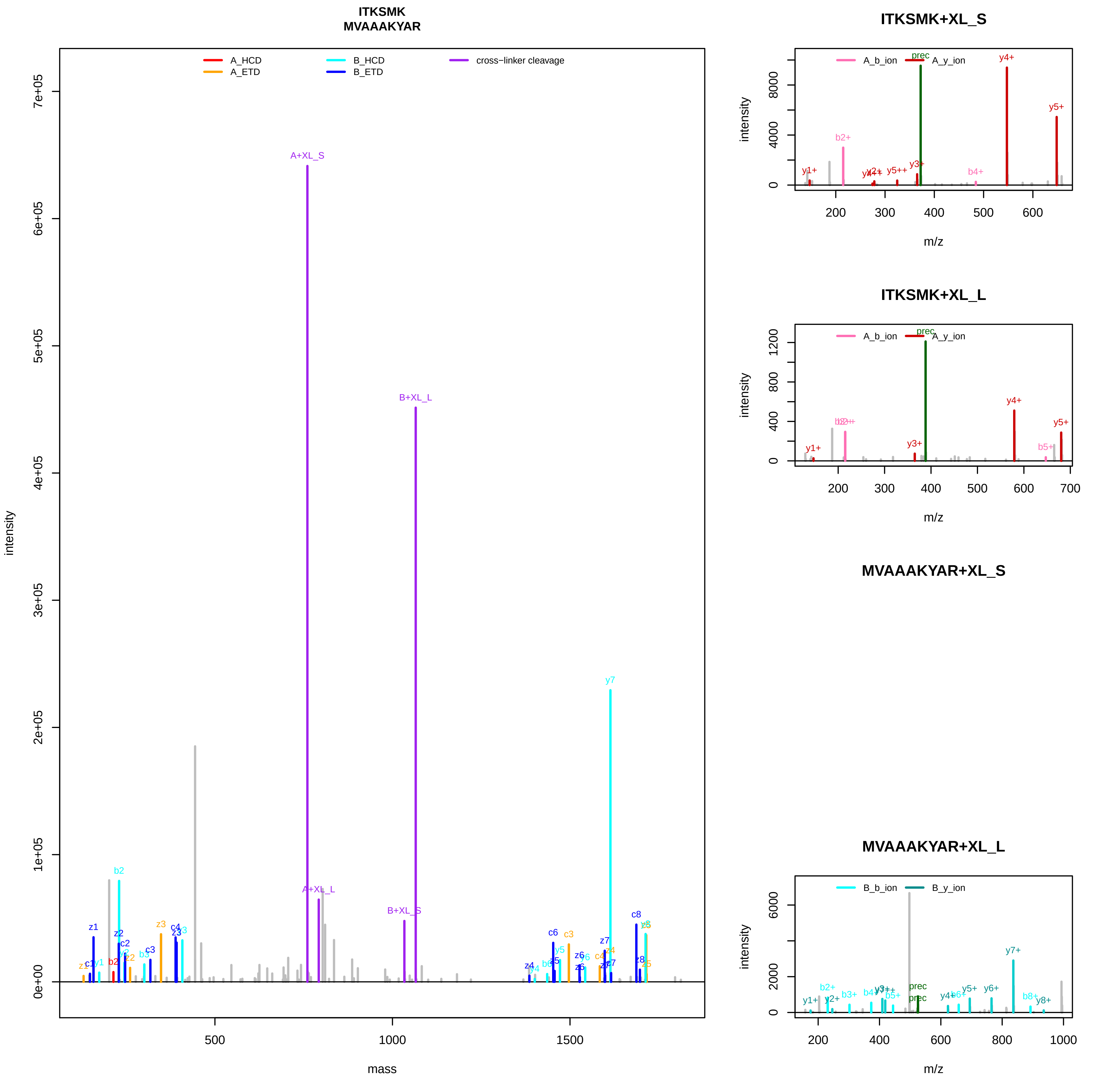
ITKSMK
MVAAAKYAR
A_HCD
A_ETD
B_HCD
B_ETD
cross−linker cleavage
A+XL_S
B+XL_L
A+XL_L
B+XL_S
y7
intensity
mass
7e+05
6e+05
5e+05
4e+05
3e+05
2e+05
1e+05
0e+00
500
1000
1500
ITKSMK+XL_S
A_b_ion
A_y_ion
prec
y4+
y5+
b2+
b4+
y1+
y3+
y5++
m/z
ITKSMK+XL_L
y4+
y5+
y3+
y1+
b5+
MVAAAKYAR+XL_S
MVAAAKYAR+XL_L
B_b_ion
B_y_ion
y7+
y5+
y6+
b8+
y8+
y1+
y2+
b2+
b3+
b5+
prec
